# Parliament and the Professions

**Published:** 1921-03-26

**Authors:** 


### Parliament and the Professions.

### Medical Staff of the Health Ministry.

Dr. Addison stated that the number of whole-time permanent and pensionable medical men employed in the Ministry of Health and the Welsh Board of Health is seventy-one.

### Medical Record Cards.

Dr. Addison informed Sir W. Davidson that the Committee set up to deal with medical record cards consists of Sir Humphry Rolleston, as Chairman, and sixteen others, fourteen being medical men, five of whom are in Insurance practice.

### Lord Cave's Hospital Committee.

Sir G. Collins asked the Minister of Health when the Report of the Committee on Hospital Finances, presided over by Lord Cave, will be published. Dr. Addison, in answering, referred to the issue of an interim report on the question of contributions to hospitals out of the valuation surpluses of approved societies. He had been informed that the evidence will be completed by the beginning of May, and the Committee hope to present their final report before the end of that month.

### Venereal Disease in the Army

The Secretary of State for War, in answer to a question, regretted to say that the incidence of venereal disease in the British Army of the Rhine is not satisfactory, and has been the subject of much anxious consideration ever since the area was first occupied. During the first quarter of 1920 the ratio admitted to hospital for venereal disease was 45.81 per 1,000 of the strength. The ratio during the last quarter of the year was 40.22, and for the month of January it was equal to a quarterly ratio of 37.80. The admission rate includes every case admitted to hospital, including relapses and readmissions for further treatment, as well as primary infections. Every effort is made to reduce the amount of disease, including the spread of information regarding self-disinfection as followed in the United Kingdom, where the incidence is now and was for the year 1920 at a lower level than at any time in the history of the British Army.

